# Direct formalin fixation induces widespread transcriptomic effects in archival tissue samples

**DOI:** 10.1038/s41598-020-71521-w

**Published:** 2020-09-02

**Authors:** Leah C. Wehmas, Susan D. Hester, Charles E. Wood

**Affiliations:** 1 Office of Research and Development, U.S. Environmental Protection Agency, MD-B105-03, 109 T.W. Alexander Drive, Research Triangle Park, NC 27709 USA; 2 Present Address: Boehringer Ingelheim Pharmaceuticals Inc., Ridgefield, CT USA

**Keywords:** RNA sequencing, Biotechnology

## Abstract

Sequencing technologies now provide unprecedented access to genomic information in archival formalin-fixed paraffin-embedded (FFPE) tissue samples. However, little is known about artifacts induced during formalin fixation, which could bias results. Here we evaluated global changes in RNA-sequencing profiles between matched frozen and FFPE samples. RNA-sequencing was performed on liver samples collected from mice treated with a reference chemical (phenobarbital) or vehicle control for 7 days. Each sample was divided into four parts: (1) fresh-frozen, (2) direct-fixed in formalin for 18 h, (3) frozen then formalin-fixed, and (4) frozen then ethanol-fixed and paraffin-embedded (n = 6/group/condition). Direct fixation resulted in 2,946 differentially expressed genes (DEGs) vs. fresh-frozen, 98% of which were down-regulated. Freezing prior to formalin fixation had ≥ 95% fewer DEGs vs. direct fixation, indicating that most formalin-derived transcriptional effects in the liver occurred during fixation. This finding was supported by retrospective studies of paired frozen and FFPE samples, which identified consistent enrichment in oxidative stress, mitochondrial dysfunction, and transcription initiation pathways with direct fixation. Notably, direct formalin fixation in the parent study did not significantly impact response profiles resulting from chemical exposure. These results advance our understanding of FFPE samples as a resource for genomic research.

## Introduction

Formalin is the most widely used fixative for biological specimens. The origins of formalin fixation date back to the early 1890s, when a young German physician named Ferdinand Blum observed that “my own fingers … became completely hardened” while studying the disinfectant properties of formaldehyde^1^. Blum promptly sent formalin-fixed tissues from a mouse to a local histology laboratory and soon after reported that these samples had less tissue shrinkage and distortion on histologic evaluation compared to standard alcohol fixation^2^ Within two years, these early observations had led to over 50 reports on formalin fixation^3^, highlighting its technical advantages and application across many tissue types. Eventually, formalin came to be the global standard for preserving most types of diagnostic and research tissue samples^4^.

The single-page report by Blum in 1893 continues to have far-reaching implications for modern science^1^. Most tissue specimens collected for pathological evaluation are processed and stored as formalin-fixed paraffin-embedded (FFPE) blocks. For many studies and clinical cases, these blocks represent the only remaining biological archive. Historically, stored FFPE samples have been used primarily for retrospective histopathological evaluations and, in some cases, biomarker development using special stains or immunohistochemistry. More recently, genomic technologies such as next-generation sequencing have greatly expanded the potential use of FFPE samples. For example, in cancer research, several large-scale collaborative efforts such as The Cancer Genome Atlas (TCGA) and the International Cancer Genome Consortium (ICGC) are exploiting FFPE samples for data mining, biomarker discovery, and related bioinformatics applications, while other efforts are using genomic profiles from FFPE RNA and DNA to subclassify different types of cancers and guide therapeutic decisions^5–7^. In toxicological science, biorepositories represent an important opportunity to streamline current approaches to risk assessment and integrate mechanistic information in a more rapid and cost-effective way. Genomic data may provide an important read-across linking early molecular effects with later health outcomes and extrapolating results across different models, including in vitro to in vivo systems. Archival FFPE samples represent an important resource for providing this type of pathway-based information without the need for additional new studies.

Our group and others have investigated mRNA profiles in paired fresh-frozen (FR) and FFPE samples and reported high concordance in fold-change values for common differentially expressed genes (DEGs)^8,9^, particularly for higher-quality FFPE samples with limited formalin fixation time and age in block. However, several distinctive features of FFPE mRNA profiles were noted, including a consistent shift in exonic reads for FFPE compared to FR samples on principal components analysis (PCA) and large sets of FFPE-related DEGs^9^. These effects were identified despite consistent increases in intronic and intergenic alignments of reads across FFPE samples and at least a 35% reduction in gene counts from FFPE samples relative to FR. Data from other microarray and RNA-sequencing (RNA-seq) studies conducted through the National Cancer Institute (NCI) and TCGA noted similar patterns in FFPE samples from different cancer types, further suggesting that formalin fixation may result in systematic transcriptomic changes^8,10–12^. This type of effect could have important repercussions for studies using FFPE samples, especially if it influences test article responses or phenotypic profiles. The main goal of this study was to investigate whether formalin fixation at the time of tissue collection induces distinctive mRNA effects in FFPE samples and, if so, how these effects may impact gene responses following chemical treatment.

## Results

**Figure 1:**
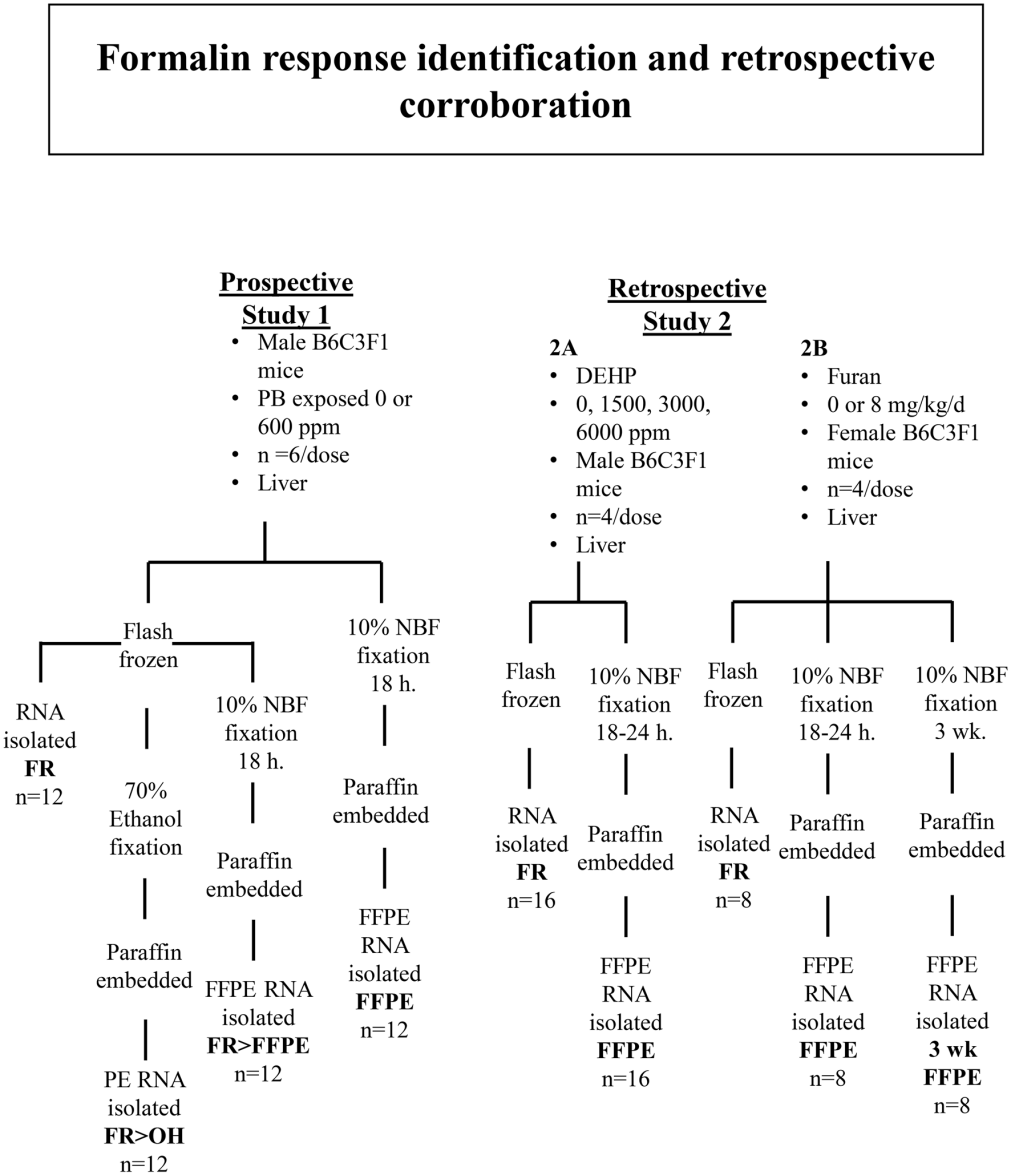
Design of experiments to prospectively (Study 1) and retrospectively (Study 2) evaluate formalin effects. *PB* phenobarbital, *DEHP* di(2-ethylhexyl) phthalate, *NBF* neutral buffered formalin, *FR* frozen, *FR* > *OH* frozen first then fixed in 70% ethanol prior to paraffin-embedding, *FR* > *FFPE* frozen first then fixed in 10% NBF for 18–24 h prior to paraffin-embedding, *FFPE* directly fixed in 10% NBF for 18–24 h or 3 weeks prior to paraffin-embedding.

Previous RNA-seq studies evaluating paired FFPE and frozen samples have shown a consistent transcriptomic bias due to FFPE preservation (e.g. Ref.^9^). We hypothesized that this shift was due to a core set of transcriptional changes that occur when fresh tissue is directly fixed in formalin. To test this idea, we conducted a series of studies comprising prospective (Study 1) and retrospective research (Study 2A and 2B) (Fig. 1).

### Formalin-induced gene expression profiles in archived tissue samples

#### Study 1

**Figure 2:**
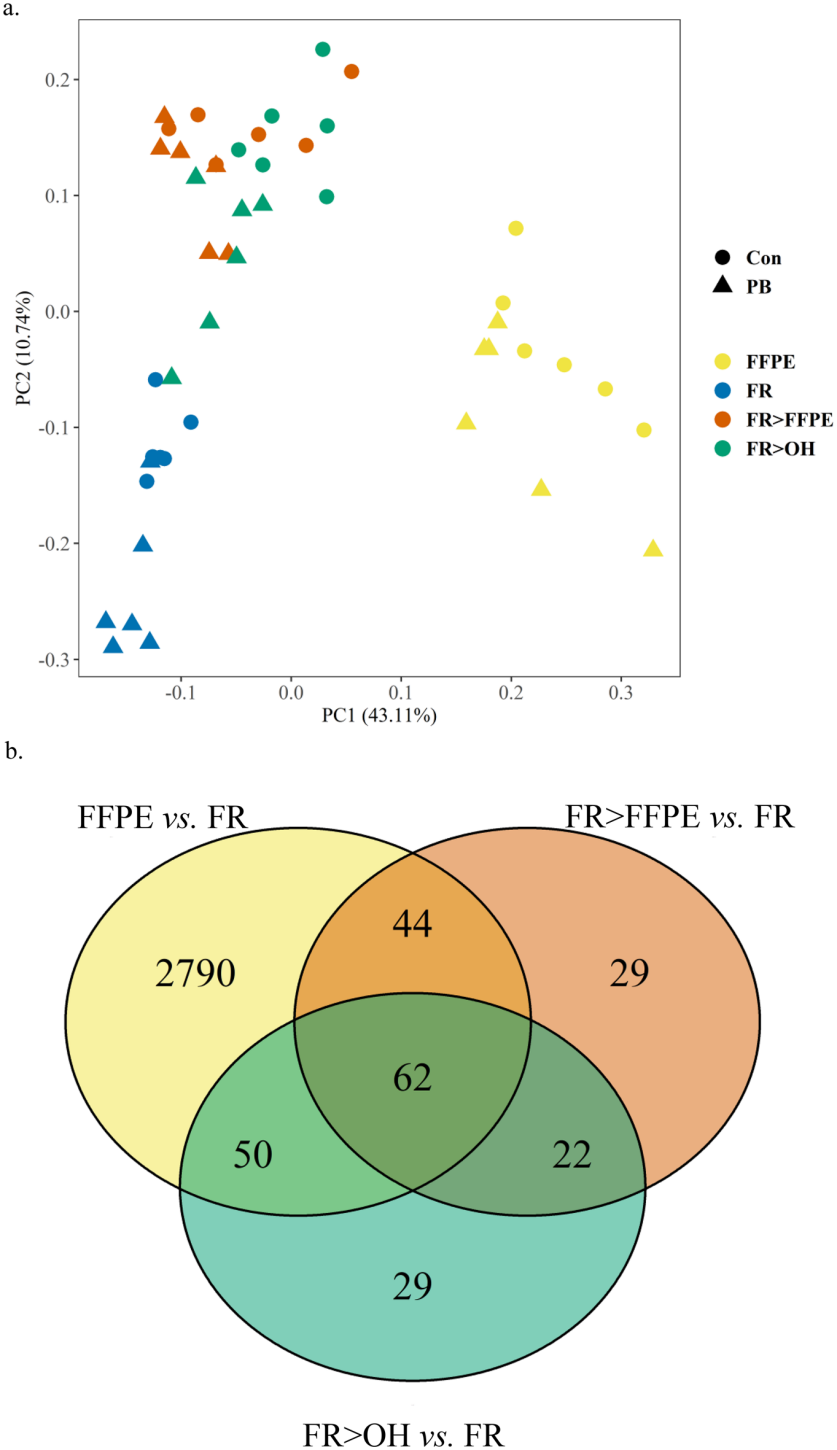
Formalin effects on gene expression by RNA-seq. (**a**) Principal component analysis on scaled, low-expression filtered, counts per million-normalized, log_2_-transformed gene counts altered by different preservation procedures. ● indicates vehicle control (Con) samples. ▲ indicates phenobarbital (PB)-treated samples. Different colors designate the preservations: frozen control (FR), 70% ethanol-fixed (FR > OH), frozen first then 18-h formalin-fixed (FR > FFPE), and 18-h formalin-fixed (FFPE) liver tissue samples. FR > OH, FR > FFPE, and FFPE samples were all paraffin-embedded. (**b**) Overlap in significant differentially expressed genes between different preservation groups relative to the paired frozen controls. Significant differentially expressed genes identified by FDR-adjusted p-value < 0.05 and fold-change cutoff ± 2 using Partek Flow Gene Specific Analysis.

Direct formalin fixation resulted in a large shift in transcriptional profiles for FFPE mouse liver samples compared to FR (Fig. 2a). Differential gene expression analysis identified that direct fixation (FFPE vs. FR) resulted in 2,946 DEGs, 98% of which were down-regulated (Supplementary Figure S1, Supplementary Table S1). The degree of downregulation was relatively modest with average fold-change of − 2.8 ± 1.1 (s.d.). Ingenuity Pathway Analysis showed the formalin effect impacts canonical pathways consistent with enrichment in oxidative stress, mitochondrial dysfunction, sirtuin signaling pathway (associated with cellular bioenergetics), and translation initiation signaling (EIF2) (Supplementary Table S2). Freezing the tissue prior to fixation in ethanol (FR > OH) or formalin (FR > FFPE) did not show the same effects of direct formalin fixation (Fig. 2b), with FR > OH and FR > FFPE samples clustering close to FR and accounting for less variance in the dataset (Fig. 2a). This trend was also reflected at the pathway and upstream regulator level, where FR > FFPE samples tended to display more similarities with FR > OH as opposed to FFPE samples based on hierarchical clustering of p-values (Supplementary Figure S2, Supplementary Table S2, S3).

Strikingly, freezing prior to fixation resulted in 94% (FR > OH vs. FR) or 95% (FR > FFPE vs. FR) fewer DEGs, indicating that the vast majority of formalin effects occurred at the time of fixation for fresh tissue only, suggesting a direct transcriptional response of the cells being fixed rather than a post-mortem artifact (Fig. 2b). Moreover, there were only 62 common DEGs across the three preservation groups compared to FR, indicating that mechanical damage associated with formalin fixation and/or tissue processing in paraffin (after fixation) had very minor effects on transcriptomic profiles in this study (Fig. 2b).

**Figure 3:**
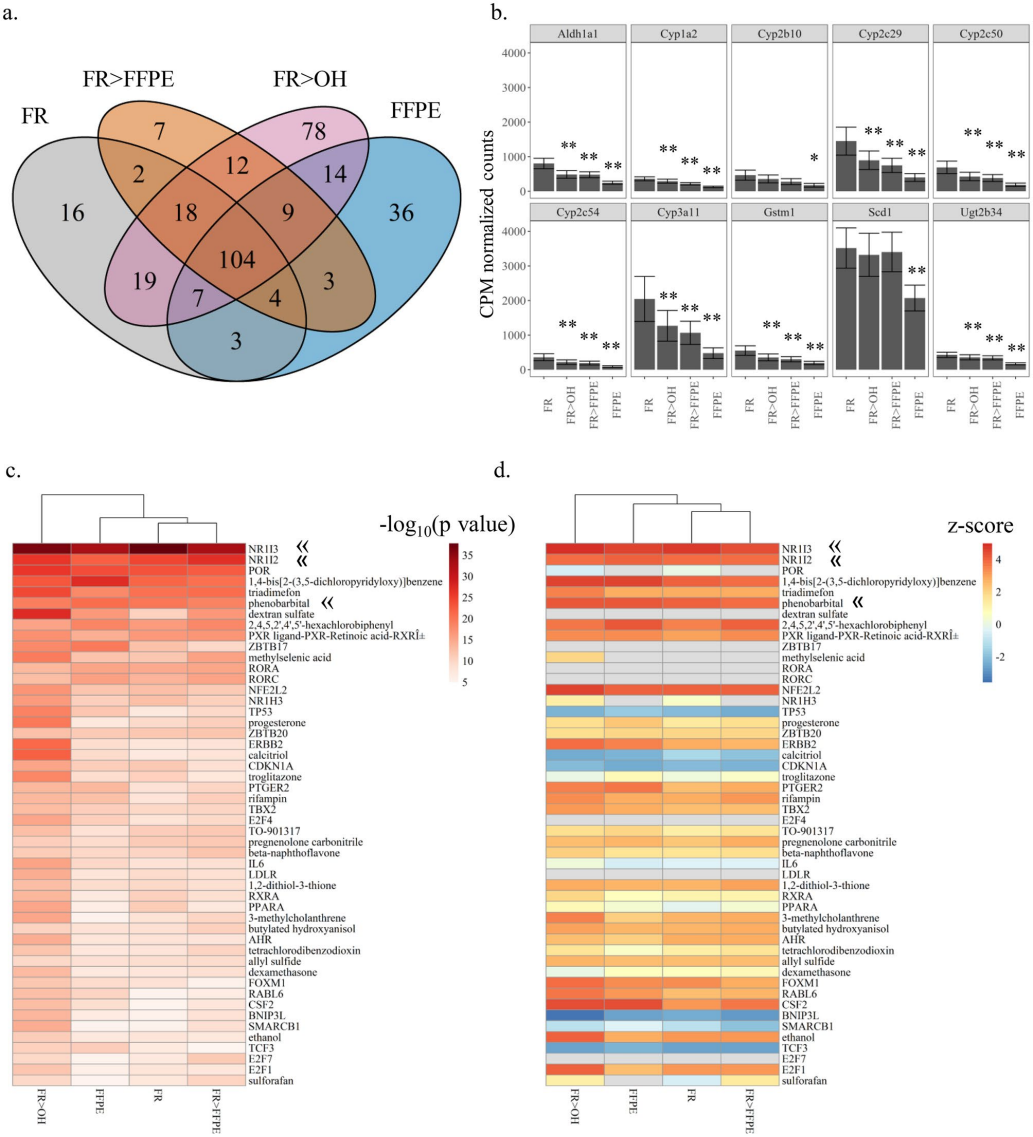
Effects of formalin fixation on detection of chemical treatment responses. (**a**) Venn diagram showing overlap in significant phenobarbital (PB)-induced differentially expressed genes (DEGs, FDR-adjusted p-value < 0.05, ± twofold-change) across preservation groups: frozen control (FR), 70% ethanol-fixed (FR > OH), frozen first then 18-h formalin-fixed (FR > FFPE), and 18-h formalin-fixed (FFPE) liver tissue samples. FR > OH, FR > FFPE, and FFPE samples were all paraffin-embedded. (**b**) Expression pattern of top 10 PB-induced differentially expressed genes. Across preservations, FR > OH, FR > FFPE, and FFPE resulted in significant reductions of counts per million (CPM) gene counts for all preservations relative to frozen control (FR). Error bars represent standard error. Significant differences between preservation group compared to frozen were determined by two-tailed Wilcoxon signed-rank test with Holm correction. **p-value = 0.0029, *p-value = 0.01 (**c**) Significant upstream regulators (Right-Tailed Fisher’s Exact Test, p-value < 0.05) predicted from PB-induced DEG patterns across preservation groups. « indicates CAR, PXR, and PB. (**d**) *Z*-scores of upstream regulators matched to significant p-values with positive *z*-scores predicting activation and negative scores predicting repression of regulator. « indicates CAR, PXR, and PB.

Despite the large effect of direct formalin fixation on global transcriptional profiles (FFPE vs. FR), it did not have a clear impact on phenobarbital (PB) responses. PB treatment induced 173 DEGs in FR, compared to 180 for FFPE, 159 for FR > FFPE, and 261 for FR > OH (Fig. 3a, Supplementary Table S4). Sixty percent of PB DEGs in FR (104) were shared with the three other preservation groups, and of those 104 DEGs only 39 were identified in common with the 2,908 formalin effect DEGs (Supplementary Figure S3). The overall pattern of DEGs changed by PB was similar across preservation groups and included well-established biomarkers of PB effect such as *Cyp2b10, Cyp2c50*, and *Cyp3a11* (Supplementary Figure S4, Supplementary Table S5). For instance, *Cyp2b10* in FR was induced 5,765-fold by PB and 1,744- to 4,111-fold across the other preservation groups. As a less dramatic example, *Aldh1a1* in FR was induced fourfold by PB and three- to fivefold in the other preservation groups (Supplementary Table S4). Because preservation-related reductions in PB and vehicle control (Con) gene counts were relatively consistent across marker genes, significant PB effects compared to Con were still detectable (Fig. 3b).

Based on the Matthew’s correlation coefficient (MCC), FR > FFPE showed the highest concordance of PB DEGs with FR at 0.77. The MCC assumes all the DEGs in FR were true positives, which is not likely the case but needed as a benchmark for assessing preservation-related changes in quality with respect to gene expression. While FR > OH appeared to have more false positives (113), this group also had fewer false negatives (25) than FFPE (55), resulting in an MCC of 0.69. The FFPE group ranked lowest amongst the preservation groups compared to FR based on MCC (0.66) but was still in positive agreement based on shared PB-induced DEGs (Table 1). The major difference in biomarker genes across preservations was that FFPE tended to have significantly fewer counts (1.7- to 4.3-fold) on average across all marker genes relative to FR (Supplementary Table S6). Freezing prior to fixation (FR > OH or FR > FFPE) also resulted in mostly significant but more moderate decreases in gene counts. For instance, FR > FFPE and FR > OH had fewer gene counts (1.3-fold to 1.9-fold and 1.2-fold to 1.7-fold and, on average, respectively) for all marker genes except *Scd1* and/or *Cyp2b10* compared to FR (Fig. 3b, Supplementary Table S6). While not significant when separated by treatment, both PB and Con groups displayed similar trends in reduced gene counts across preservations relative to FR (Supplementary Figure S5, Supplementary Table S7).

**Table 1:** Significant phenobarbital (PB)-induced changes in gene expression compared to control (Con) confusion matrix across preservations.

	DEGs	TP	FP	FN	TN	MCC
FR^a^	173	173	0	0	13,197	1.00
FR > OH	261	148	113	25	13,084	0.69
FR > FFPE	159	128	31	45	13,166	0.77
FFPE	180	118	62	55	13,135	0.66

DEGs identified by FDR-adjusted p-value < 0.05 and absolute fold-change value ≥ 2. MCC coefficient = 1 represents a perfect prediction; 0 represents no better than random; -1 indicates disagreement between observation and prediction.

*DEGs* differentially expressed genes, *TP* true positives, *FP* false positives, *FN* false negatives, *TN* true negatives, *MCC* Mathews correlation coefficient.

^a^Assuming FR control contains the actual PB-induced DEGs; which may not be the case.

When PB DEGs were mapped to potential upstream regulators, gene patterns were consistent with enrichment and predicted activation of constitutive androstane receptor (CAR) and pregnane X receptor (PXR) and PB exposure across all preservation groups (Fig. 3c,d and Tables S8, S9). At this pathway/functional level, no impact of direct formalin fixation on chemical response was observed.

### Retrospective comparison of formalin-induced gene expression profiles in archived tissue samples

#### Study 2

A retrospective analysis of previous experiments comparing RNA-seq profiles from paired FFPE and FR mouse liver tissue^8,9^ also identified a robust FFPE effect at the transcript level (Supplementary Figure S6a). While there were broad patterns in formalin effect across examined studies, the DEGs were not consistent. Direct formalin fixation for 18 h. resulted in 2,283 DEGs in Study 2A and 3,525 DEGs in Study 2B (Supplementary Figure S6b, Supplementary Table S10). In Study 2A, 163 DEGs were identified in all dose groups. In Study 2B, there were 1,277 shared formalin DEGs across both treatment and control groups. With the inclusion of the 3 week timepoint, formalin DEGs in Study 2B increased to 5,212. FFPE samples fixed for 3 week had 3.5-fold more formalin DEGs compared to FFPE samples fixed for 18 h., suggesting an influence of time in formalin likely linked to increased physical damage to the nucleic acid (Supplementary Table S10). However, when focusing on the overlap in formalin DEGs across Study1, Study2A, and Study2B from the 18–24 h. formalin fixation timepoint, there was still a high level of overlap regardless of chemical treatment. For example, when focusing on vehicle control samples, 75% of FFPE vs. FR DEGs in Study 2B overlapped with FFPE vs. FR DEGs from Study 1 controls (1,379/1851) and 61% of FFPE vs. FR DEGs in Study 2A overlapped with FFPE vs. FR DEGs in Study 2B (322/530) (Supplementary Figure S6c). The percent overlap increased when considering DEGs in common for chemical treatment samples and vehicle controls for FFPE vs. FR (Supplementary Figure S6c).

Pathway analysis identified enrichment in similar canonical pathways between directly fixed samples from Studies 1 and 2 (Supplementary Figure S7a) including mitochondrial dysfunction, oxidative phosphorylation, sirtuin signaling, and EIF2 signaling (Eukaryotic Translation Initiation Factors) as some of the top impacted pathways (Supplementary Table S11). This was also the case for predicted upstream regulators, some of which also happen to be regulators of oncogenesis such as MYC, MYCN, and INSR. The formalin effect overlapped with gene signatures of some drug/chemical compounds including pirnixic acid, ciprofibrate, gentamicin, and mono(2-ethylhexyl) phthalate, the primary metabolite of DEHP. Freezing prior to preservation in Study 1 mostly negated the gene response profile for these predicted upstream regulators (Supplementary Figure S7b, Supplementary Table S12).

When focusing on individual molecules of FFPE vs. FR impacted pathways, effects on mitochondrial dysfunction and oxidative phosphorylation included downregulation of several components in the electron transport chain such as multiple ATP synthase subunits, ubiquinone oxidoreductase subunits, and ubiquinol-cytochrome c reductase, which were not impacted in FR > FFPE or FR > OH samples (Supplementary Tables S13 and S14). Effects specific to direct formalin fixation (FFPE vs. FR) on the sirtuin signaling pathway and EIF2 signaling included genes related to controlled cell death, RNA synthesis, DNA damage recognition, and translation processes (Supplementary Tables S15 and S16).

As in Study 1, direct formalin fixation reduced overall gene counts across control and treated groups but had minimal impact on detection of DEHP (2A) and Furan (2B) effects at gene and pathway levels. For example, of the 163 and 1,173 formalin effect DEGs from Studies 2A and 2B, respectively, only 8 DEGs overlapped with DEHP treatment genes and 27 DEGs overlapped with Furan treatment genes (Supplementary Figure S8). Additional analysis of the chemical treatment gene response from Studies 2A and 2B are reported in detail elsewhere^8,9^. While the RNA-seq analysis pipelines for Studies 2A and 2B differed slightly from those reported in Hester et al*.* and Webster et al*.*, a concordance in chemical treatment responsive genes and pathways was observed for each respective study.

## Discussion

The primary goal of this study was to investigate the effects of formalin fixation on gene expression profiles in archival FFPE samples. Our findings clearly demonstrate that direct formalin fixation results in widespread transcriptomic effects, as detected by RNA-seq analysis. We show for the first time that formalin effects on total RNA-seq profiles occur at the time fresh liver tissue is fixed and that this effect is largely abolished by freezing first, suggesting the gene response to formalin is in part a biological response and not simply post-mortem artifact. Gene profiles resulting from direct fixation indicated disruption of fundamental pathways of cell metabolism, including oxidative stress, mitochondrial dysfunction, and bioenergetic pathways. However, these effects did not confound the detection of chemical treatment-related responses. These findings could have important implications for studies using FFPE samples, especially if adequate matched controls are not available.

Sequencing analysis of FFPE specimens provides an opportunity to study samples from well-characterized archives, ranging from toxicology repositories^13^ to clinical tumor biobanks^14,15^. At the gene level, direct formalin fixation resulted in a broad low-magnitude effect on global transcriptomic profiles. Several lines of evidence suggest that this response may be due to disruption of cellular processes during fixation of fresh cells. First, the effect was eliminated by freezing the tissues prior to fixation in Study 1. Second, FFPE DEGs showed widespread down-regulation of expression on RNA-seq analysis, as expected with formaldehyde-induced cross-linking. Third, pathway-level and functional analysis of FFPE DEGs showed disruption of key metabolic functions related to cellular respiration, mitochondrial toxicity, and bioenergetics, which are consistent with agonal effects of cells accumulating formaldehyde crosslinks. Finally, formaldehyde is a ubiquitous endogenous metabolite of many different cell types, which have evolved sensitive mechanisms to detect and respond to it^16^.

Another recent study described formalin responses on RNA-seq profiles while investigating the impact of delay-to-fixation on FFPE clinical tumor resections^17^. Interestingly, the act of preservation for standard FFPE samples, regardless of delay-to-fixation times, had a larger impact on gene response when compared to fresh-frozen samples. Jones et al*.* also reported an upregulation of genes in FFPE RNA that was consistent with structural DNA modifications, as opposed to the metabolic effects identified in the present study^17^. This discrepancy could be partly due to differences in tissues analyzed (e.g. tumor vs. normal) and/or differences in tissue type or species investigated. Cancerous tissues are notoriously heterogeneous and exhibit altered RNA profiles relative to normal tissues, including genes related to cellular bioenergetics, potentially obscuring the relatively low-magnitude formalin effects described in the current analysis. Whatever the case, these studies highlight the importance of preanalytical variables in determining the response of tissues to formalin fixation and warrant additional work to determine whether similar effects are seen across different tissue types and species under different study conditions.

The observation of global transcriptomic effects due to formalin fixation is not entirely unexpected considering the dynamics of tissue preservation. Most importantly, covalent binding of formaldehyde within cells of fixed tissue does not occur immediately. The chemical modifications of RNA, including cross-linking, can continue for up to 24 h following immersion of a tissue^4,18,19^. Formalin penetrates tissue quickly, but a lag in tissue penetration of formalin into the middle of a specimen may still result in some cells remaining unfixed or underfixed for minutes or even hours after initial immersion. This lag may provide sufficient time-to-cell death for agonal transcriptomic effects to occur. However, several studies, including Study 2B and others^9^, also indicate that age in FFPE block can have a marked impact on RNA-Seq profiles. Collectively, this evidence suggests that a combination of events is occurring, direct “agonal” effects at the time of fixation as well as non-specific RNA degradation with time.

The downregulation in genes identified by RNA-seq in FFPE tissue from mouse liver is consistent with formalin fixation-related covalent modifications of nucleic acid. RNA-seq involves sequencing all isolated RNA transcript pieces including exons, introns, and intergenic regions. Pre-mRNA or partially processed transcripts will be detected using this method, which may explain why previous work identified a modest decrease in percent reads fully aligned to exonic regions of the transcriptome from more recently archived FFPE samples compared to paired FR with a corresponding increase in the percent of intronic and intergenic alignments^9,20^. Other groups have identified a similar pattern when comparing FFPE to FR samples (e.g. Refs.^8,17^). The cause for a higher percentage of intronic reads in FFPE compared to FR is not well characterized but could be due to a variety of formalin effects on library preparation and specific cellular processes, such as having proteins bound to intronic regions, resulting in interference with transcription and translation. This disruption can impact the detection of genes in FFPE samples compared to FR samples because reads are diverted to intronic and intergenic regions. Newer technologies such as TempO-sequencing may be able to bypass part of this problem through the use of short probes that bind to specific exonic regions within genes, maintaining greater specificity^21^.

The large transcriptional effects of formalin fixation did not confound detection of chemical responses. Despite some loss in gene count signal across chemical treated and control samples in FFPE tissue samples from Study 1, well known marker genes (*Cyp2b10*, *Cyp3a11*, *Cyp2c50*, etc.) were identified and DEGs were mapped to known receptors involved in PB mode of action like CAR and PXR. This finding was also observed and previously reported for Study 2A^9^ and 2B^8^ and their respective chemical treatments. When contrasting chemical treatment vs. vehicle control, Hester et al. reported FFPE samples demonstrated 31–45% loss in gene counts relative to paired FR but still showed a significant dose-dependent upregulation of DEHP target genes *Acot* and *Cyp4a* and pathway-level enrichment consistent with peroxisome proliferator-activated receptor-alpha (PPARα)^9^. In Webster et al*.*, DEGs identified in FFPE from contrasting furan chemical treatment vs. vehicle control resulted in overrepresentation in Nrf2-mediated oxidative stress response, xenobiotic metabolism, and p53 signaling pathways, consistent with the furan mode of action^8^. However, with Study 1 there were potential spurious PB-related DEGs in the FFPE dataset that were absent in FR. These potential false-positive DEGs highlight the need for matched disease/normal or chemical/vehicle controls when working with gene expression data from FFPE tissue samples. More research is needed to better develop methods that may assist in informatically subtracting out such formalin effects when matched FFPE controls are not available.

Current sequencing technologies provide unprecedented opportunities to bridge genomic information with high-quality phenotypic data from toxicological and clinical biorepositories. However, our understanding of formalin fixation and how it interacts with new methods such as sequencing in liver remains quite limited. This study provides new insights into formalin effects on RNA-seq data from liver archival tissues. While our results highlight potential widespread effects of direct formalin fixation (and marked variability across studies), they also show that, with adequate controls, FFPE samples can be effectively used to produce reliable transcriptomic data. These findings should inform retrospective mechanistic investigations and precision medicine initiatives using archival tissue specimens.

## Methods

### Experimental overview

#### Study 1: prospective investigation of direct formalin fixation on transcriptomic profiles in mouse liver

Paired frozen and FFPE mouse liver samples used for this study were originally collected from 10- to 11-week old male B6C3F1 mice treated daily for 7 days with 600 ppm phenobarbital (PB) (99.8% purity, lot# SLBF7347V; Sigma-Aldrich, St. Louis, MO) or vehicle control (0 ppm, Con) in the drinking water (n = 6 mice/treatment group). Complete details of this study are described in Lake et al*. *^22^. All procedures involving animals in this study were conducted under protocols approved by the Institutional Animal Care and Use Committee of the U.S. EPA. Phenobarbital was used as a reference chemical given its well-characterized transcriptomic effects in mouse liver (e.g.^23^.).

Each liver tissue sample was processed four different ways (Fig. 1). At the time of sample collection, the tissue was either flash frozen in liquid nitrogen followed by storage at − 70 to − 80 °C or directly fixed in 10% neutral buffered formalin (NBF) for ~ 18 h prior to embedding in paraffin block (FFPE group). Fresh 10% NBF (ThermoFisher Scientific; cat. #SF100-4; Fairlawn, NJ) contains approximately 4% formaldehyde (weight/volume). Frozen and fixed mouse liver samples included pieces of left lateral, caudate, and right medial liver lobes. Frozen liver samples were later divided on dry ice into three portions (~ 20–30 mg each), which were returned to − 80 °C storage (FR), fixed for ~ 18 h in 70% ethanol followed by paraffin-embedding (fixative control, FR > OH), or fixed for ~ 18 h in 10% NBF followed by paraffin-embedding (FR > FFPE) (n = 12 mice per condition, 6 Con and 6 PB-treated).

Histologic processing and sectioning of FFPE, FR > OH, and FR > FFPE samples followed standard methods^20^. Briefly, tissues were washed two times in 80% ethanol (30 min each); two times in 95% ethanol (45 min each); three times in 100% ethanol (45 min each); three times in xylenes (ThermoFisher Scientific, Fairlawn, NJ) (30 min each); two times in Paraplast Tissue Embedding Media (ThermoFisher Scientific, Fairlawn, NJ) at 58–60 °C (30 min each); and, finally, two times in Paraplast at 58–60 °C under a vacuum (1 h each). After processing, FFPE blocks were stored in a research laboratory at room temperature. At the time of RNA isolation, tissue age in block was ~ 16 months.

While tissue cross-linking by formaldehyde begins almost immediately upon exposure^4^, the specific timing of formalin effects on biological processes such as RNA transcription is not understood. Freezing prior to fixation was intended to test whether observed the transcriptomic effect of formalin fixation occurs as an agonal event (i.e. during cell death, when certain aspects of metabolic and transcriptional machinery may still be operational) or as a post-mortem artifact (i.e. after cell death has occurred and transcription has stopped). Ethanol was used as a fixative control as it does not produce the cross-links and other covalent modifications seen with formalin fixation^4^.

#### Study 2: retrospective evaluation of formalin fixation on transcriptomic profiles in mouse liver

For Study 2A, paired frozen and FFPE mouse liver samples were collected from male B6C3F1 mice treated with di(2-ethylhexyl) phthalate (DEHP) (CAS No: 117-81-7; 99.5% purity, Chem Service, West Chester, PA) in the feed for 7 days at dietary doses of 0; 1,500 (1.5 k); 3,000 (3 k); and 6,000 (6 k) ppm. Experimental design and background information are described in Lake et al*.* and Hester et al*.*^9,22^. Each liver sample was divided and directly flash frozen in liquid nitrogen followed by storage at − 70 to − 80 °C (FR) or directly fixed in 10% NBF for 18–24 h followed by processing to FFPE block. A total of 16 sample pairs were used (n = 16 FR, n = 16 FFPE, n = 4 per dose group). After histologic processing, FFPE blocks were stored at ambient temperature for < 2 years. For Study 2B, paired frozen and FFPE mouse liver were collected from female B6C3F1 mice exposed to 0 or 8 mg/kg furan in corn oil by daily gavage for 3 wks. Experimental design and background information are described in Webster et al*.*^8^. At collection, each liver sample was divided and directly flash frozen in liquid nitrogen and maintained at ≤ − 70 °C or fixed in 10% NBF for 18 h. or 3 wks. before processing to FFPE block. A total of 8 sample pairs were used (n = 8 FR, n = 8 FFPE 18 h., n = 8 FFPE 3 wks., at n = 4 per dose group). FFPE blocks were stored at ambient room temperature for < 1 yr. Note: Ischemia time was not investigated in these studies. Sample collection was well controlled and time to freezing or fixation did not exceed 15 min for any samples, in accordance with recommendations to limit ischemia time to < 12 h. for FFPE samples undergoing RNA analysis^23,24^.

### RNA isolation

For frozen tissue samples, total RNA was extracted and purified using homogenization in RNAzol RT (Molecular Research Center, Cincinnati, OH) and elution with RNeasy MinElute columns (Qiagen GmbH, Hilden, Germany)^25^. The RNA quality of each sample was evaluated by Agilent 2100 Bioanalyzer and quantitated via Nanodrop and/or Qubit fluorometer (ThermoFisher Scientific, Waltham, MA). For FFPE samples, blocks were first sectioned into two 10-μm thick curls per sample using a Leica RM 2155 microtome (Leica Biosystems, Buffalo Grove, IL). RNA was then extracted following deparaffinization, proteinase K digestion (56 °C for 15 min), heating (80 °C for 15 min), DNAse treattment, RNeasy MinElute spin column (Qiagen GmbH, Hilden, Germany) clean up, and elution in 20–30 μl nuclease free water according to Qiagen AllPrep DNA/RNA FFPE kit protocols. RNA quality and concentration were quantified as for frozen samples. Quality parameters for input RNA and RNA-seq were evaluated for all samples. Values for select metrics are presented by study and sample type in Supplementary Data. Pre-RNA sequencing quality metrics for Study 2A and Study 2B are reported in Hester et al.^9^ and Webster et al.^8^, respectively.

### Total RNA-sequencing library preparation and sequencing

For RNA-seq, 100 ng of total RNA was converted into cDNA libraries using the Illumina TruSeq Stranded Total RNA, as previously described (Wehmas et al*.*^20^ for Study 1; Hester et al*.*^9^ for Study 2A; Webster et al*.*^8^: Study 2B). Ribosomal RNA was removed from samples through sequence-specific rRNA depletion using biotinylated probes and strepavadin bead immobilization (Ribo-Zero Gold Library Prep Kit, #RS-122-2303, Illumina, San Diego, CA). Samples were fragmented by heating with divalent cations. FFPE samples were subjected to reduced fragmentation/heating times to enable consistent library size distributions with FR. This process was quantified by Agilent Bioanalyzer (DNA 1000 kit #5067-1504) and qPCR (KAPA Library Quant Kit #KK4824, KAPA Biosystems, Wilmington, MA) followed by library normalization and sequencing. Samples were labeled with a barcode, mixed together in a sequencing pool, and run at eight per sequencing lane. Sequencing for Study 1, Study 2A, and Study 2B was performed at Expression Analysis (EA) (EA Genomic Services, Q2 Solutions—a Quintiles Quest Joint Venture, Durham, NC) using the Illumina HiSeq platform with 2 × 50 bp-paired, 2 × 50 bp-paired, and 1 × 50 bp-single end reads, respectively. Samples for these studies were run in independent batches.

### Sequencing analysis

After sequencing, basecall files were converted into FASTQ output files using CASAVA (1.8.2). FASTQ data were demultiplexed, and sequencing adapters and other low-quality bases were removed from the ends of reads during clipping and trimming using the fastq-mcf3 tool (available at https://github.com/ExpressionAnalysis/ea-utils/blob/wiki/FastqMcf.md) (Study 1 and 2A) or Partek Flow (Study 2B). For Studies 1 and 2A, trimming included removal of Illumina adapters, homopolymers at read ends and nucleotides with quality scores (Phred Q-scores) < 7. Any read with one base > 95% frequency, homopolymers ≥ 4 within a read, and an average Q-score below 25, or length < 25 bases were also filtered by fastq-mcf3 tool. For Study 2B, read ends were trimmed by quality (Q-scores ≤ 7) and reads with length ≤ 25 or composed of any one base at > 95% frequency were removed by Partek Flow.

Total RNA-seq reads were aligned to External RNA Controls Consortium (ERCC) spike-ins to assess the success of library construction and sequencing. A subset of the reads (~ 1 million) was aligned to other added control sequences (PhiX and other Illumina controls used during library preparation), residual sequences (globin and rRNA), and poly-A/T sequences that persisted after clipping. Reads were also aligned to a sampling of intergenic regions to assess the level of DNA contamination.

Subsequent data analysis was carried out using Partek Flow NGS v 6.17.1128 (Partek Inc., St. Louis, MO). Total RNA-seq reads were aligned using STAR v2.5.3a and counts matrices were generated using the Expectation–Maximization algorithm^26^ implemented in Partek Flow. Clipped FASTQ files were aligned to the *Mus musculus* reference genome (GRCm38/mm10) and quantified to the transcriptome (RefSeq transcript 81-2017-05-02). A 0.0001 offset was added adjust for zero counts. Gene features with geometric mean of ≤ 1 count across all samples were filtered prior to counts per million normalization (CPM). This involves dividing feature counts by total sample library size and multiplication by 10^6^. Low expression filtering to exclude unexpressed or lowly expressed gene features across all samples which may interfere with downstream differential gene expression analysis removed 11,173/24,543 gene features for Study 1; 11,107/24,543 gene features for Study 2A; and 11,368/24,543 gene features for Study 2B. Filtered, normalized gene counts were then analyzed for differential gene expression using Partek Gene-Specific Analysis algorithm (GSA). Significance was defined as false discovery rate (FDR)-adjusted p-value of < 0.05 and absolute fold-change count ≥ 2.

### Other statistical analyses

Other data were analyzed using R statistical software (v3.5.3)^27^, *pheatmap* (v1.0.12), *VennDiagram* (v1.6.20), *ggplot2* (v3.1.1), *stats* (v3.5.3), and *car* (v3.0-2) packages. Continuous data were first screened for normality using the Shapiro–Wilk Test and homogeneity of variance using the Levene's Test. As most statistical comparisons for the sequencing quality metrics did not meet normality or homogeneity of variance criteria, all data were analyzed using non-parametric two-tailed Wilcoxon signed-rank tests with a Holm multiple comparison adjustment. An adjusted p-value < 0.05 was the threshold for significance. To assess the effects of chemical exposure (Study 1), phenobarbital treatment was contrasted to the respective control within the same preservation condition (i.e., FR, FR > FFPE, FFPE, etc.). Where chemical treatment and preservation interactions were not expected or observed for pre-sequencing, pre-alignment, and preanalytical comparisons, PB and Con samples were combined for analyses. Pairwise comparisons included each preservation group vs. FR control.

## Supplementary information

Supplementary file 1

Supplementary file 2

Supplementary file 3

Supplementary file 4

Supplementary file 5

Supplementary file 6

## Data Availability

RNA-sequencing and TempO-sequencing datasets generated during the current study are available at Gene Expression Omnibus (https://www.ncbi.nlm.nih.gov/geo/): Study 1 accession # #GSE148174, Study 2A accession #GSE78962 and CEBS #010-00006-0001-000-8, and Study 2B accession #GSE62843). All other data created or analyzed during this study are included in this published article.

## Abbreviations

CAR: Constitutive androstane receptor
DEG: Differentially expressed gene
EA: Expression analysis
EPA: U.S. Environmental Protection Agency
ERCC: External RNA controls consortium
FDR: False discovery rate
FFPE: Formalin-fixed paraffin-embedded
FR: Fresh-frozen
GSA: Gene-specific analysis
ICGC: International cancer genome consortium
IPA: Ingenuity pathway analysis
MCC: Matthew’s correlation coefficient
MOA: Mode of action
qPCR: Quantitative polymerase chain reaction
PXR: Pregnane X receptor
NCI: National Cancer Institute
OH: Ethanol
PB: Phenobarbital
PPARα: Peroxisome proliferator-activated receptor-alpha
PCA: Principal component analysis
RIN: RNA integrity number
RNA-seq: RNA-sequencing
rRNA: Ribosomal RNA
STAR: Spliced transcripts alignment to a reference
TCGA: The cancer genome atlas
