# Supplementary material for: Direct formalin fixation induces widespread transcriptomic effects in archival tissue samples
Source: Sci Rep. 2020 Sep 2;10:14497. doi: 10.1038/s41598-020-71521-w (PMC7468282; doi:10.1038/s41598-020-71521-w)
Supplement: Supplementary file 5 [file 41598_2020_71521_MOESM5_ESM.pdf]

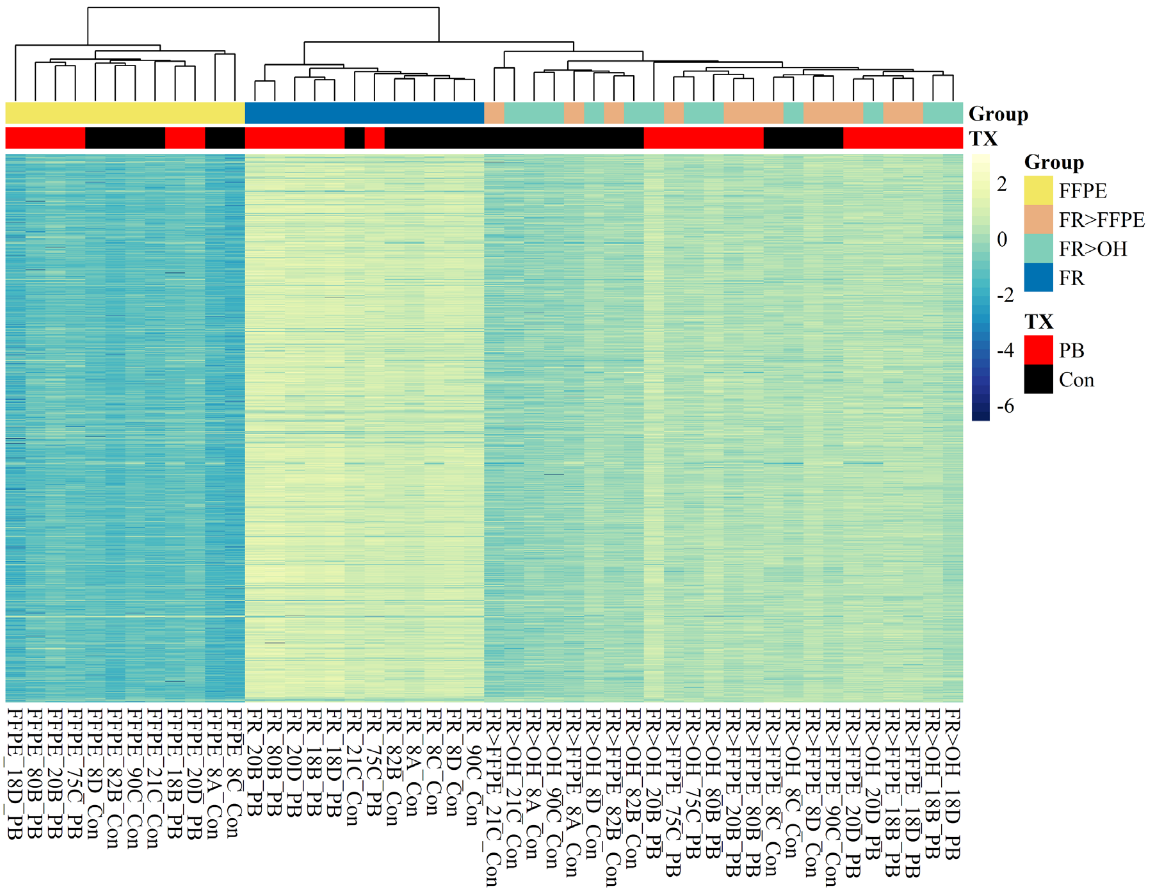

Supplementary Figure S1

a.

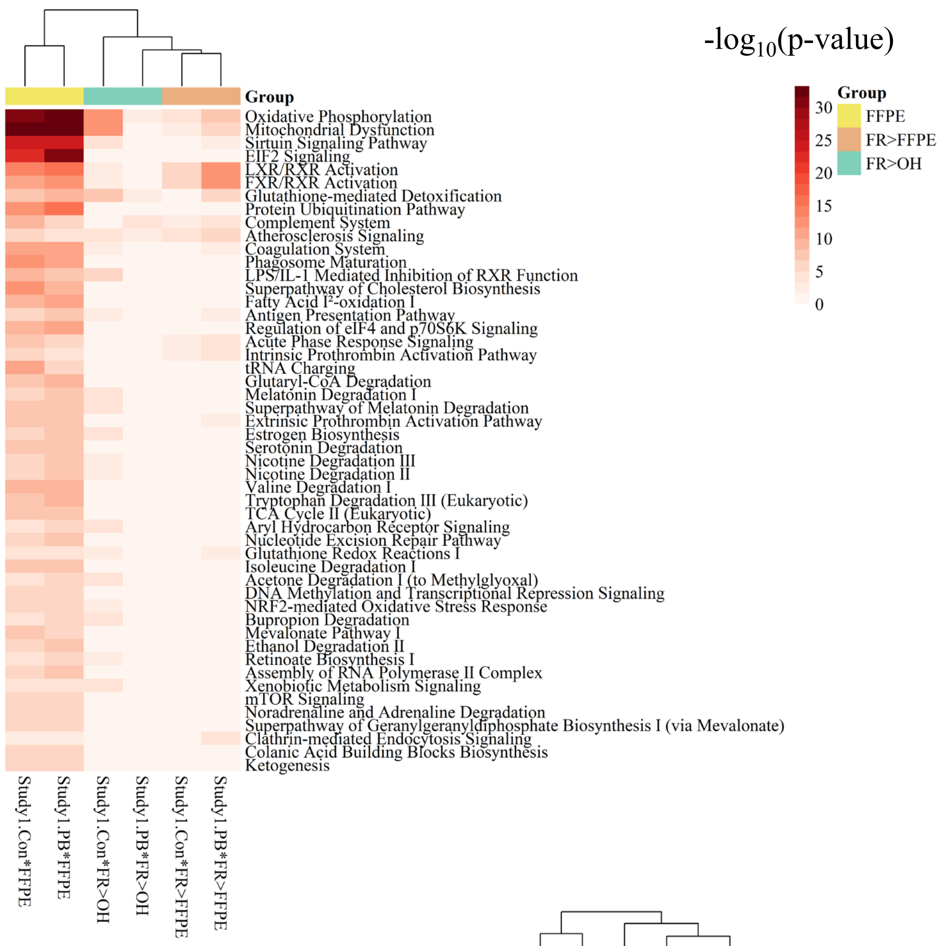

b.

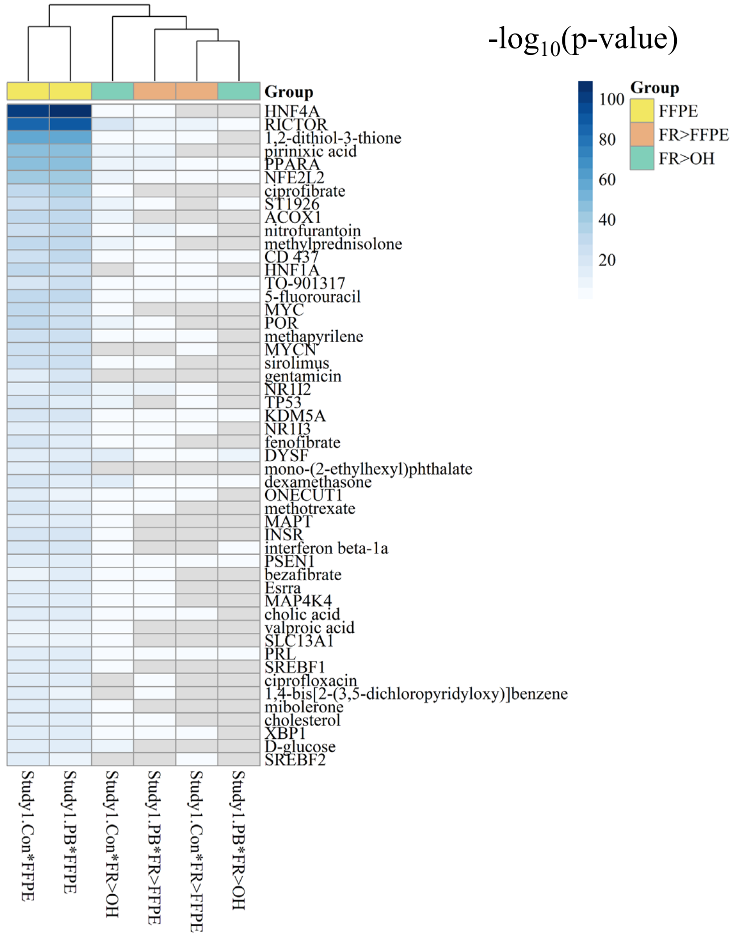

Supplementary Figure S2

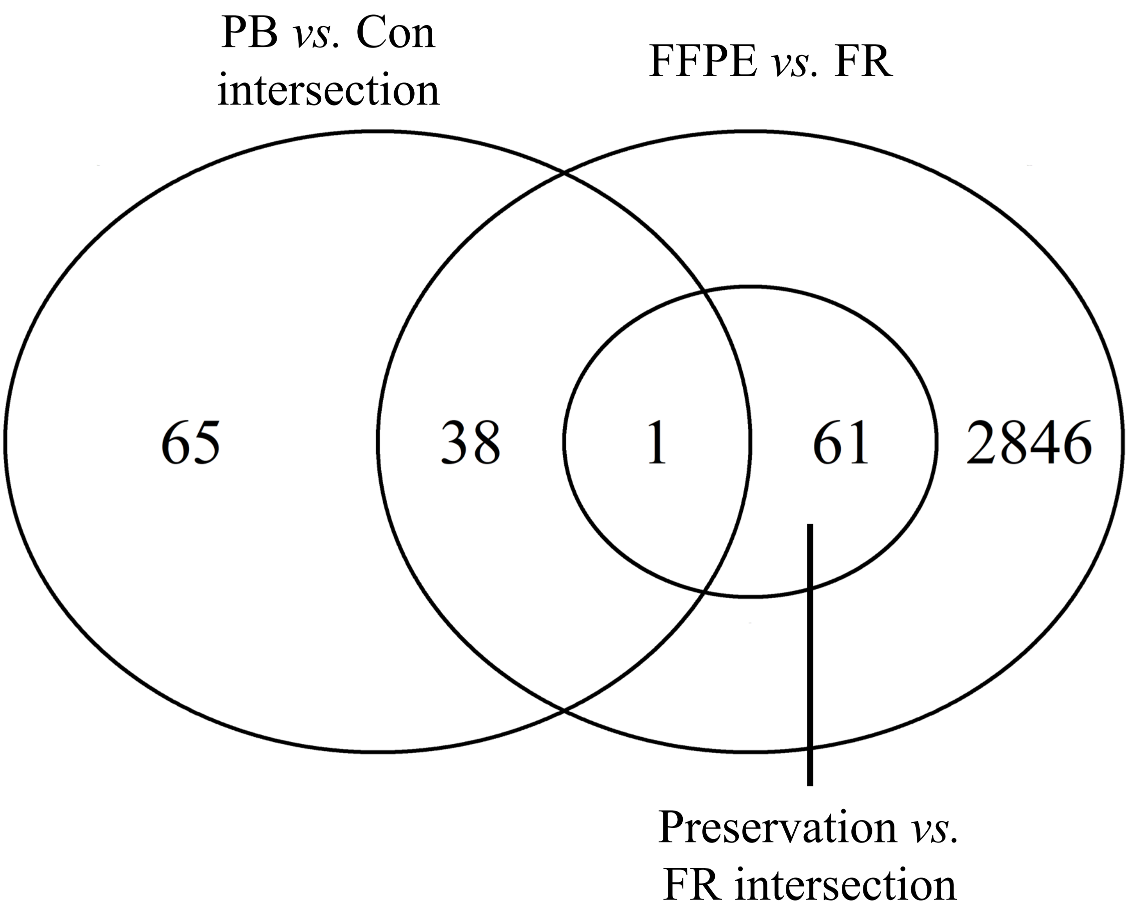

Supplementary Figure S3

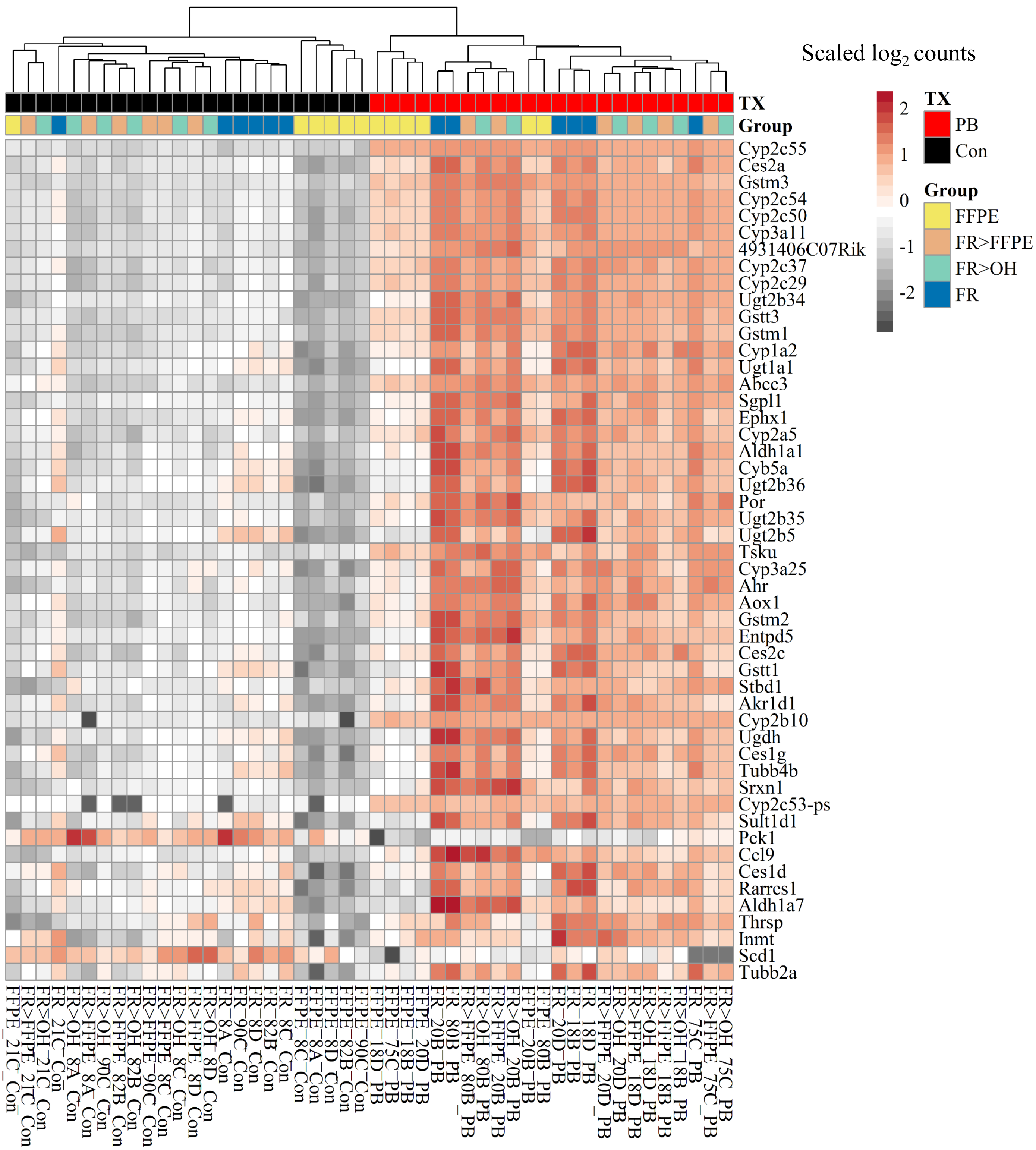

Supplementary Figure S4

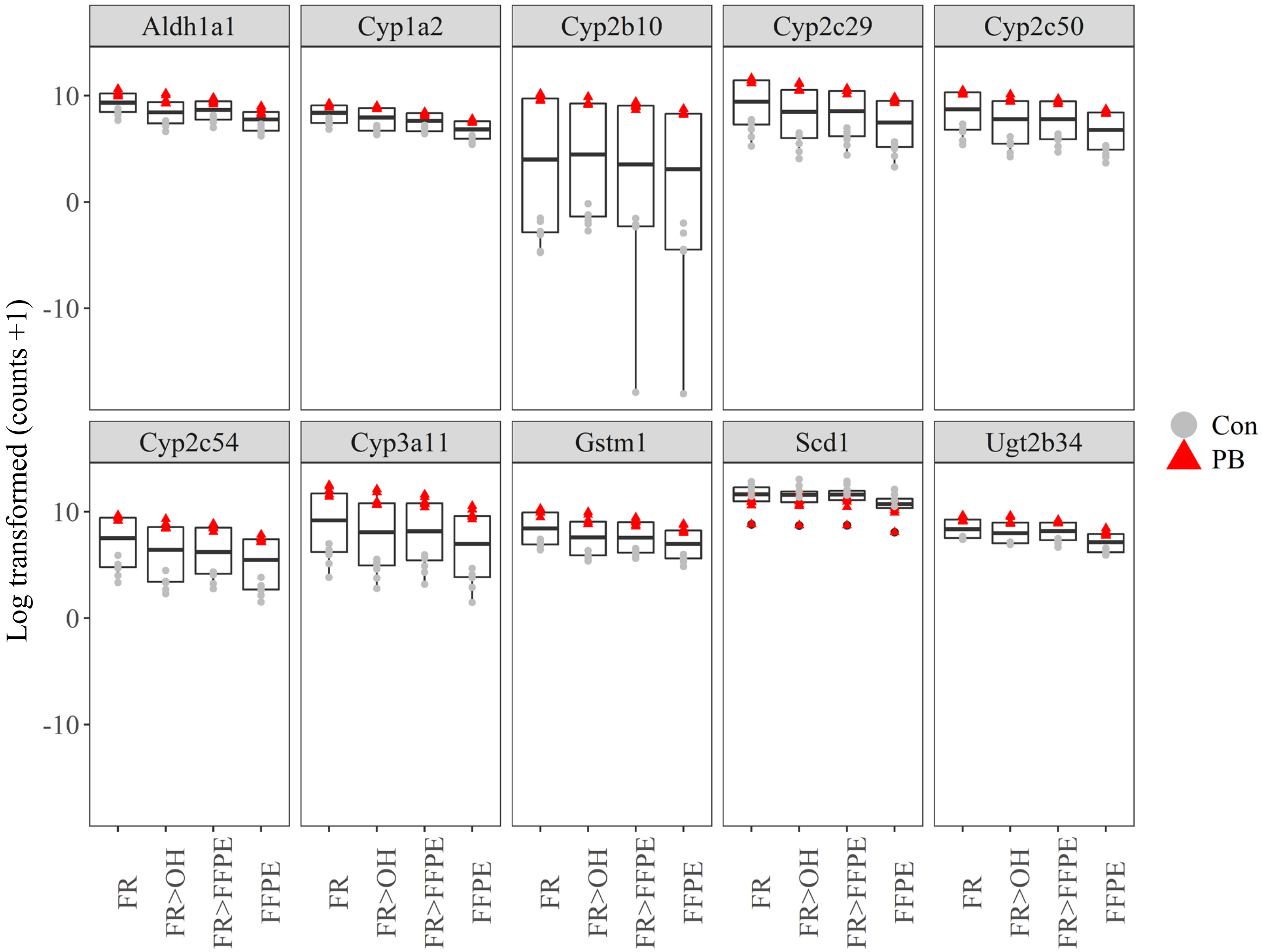

Supplementary Figure S5

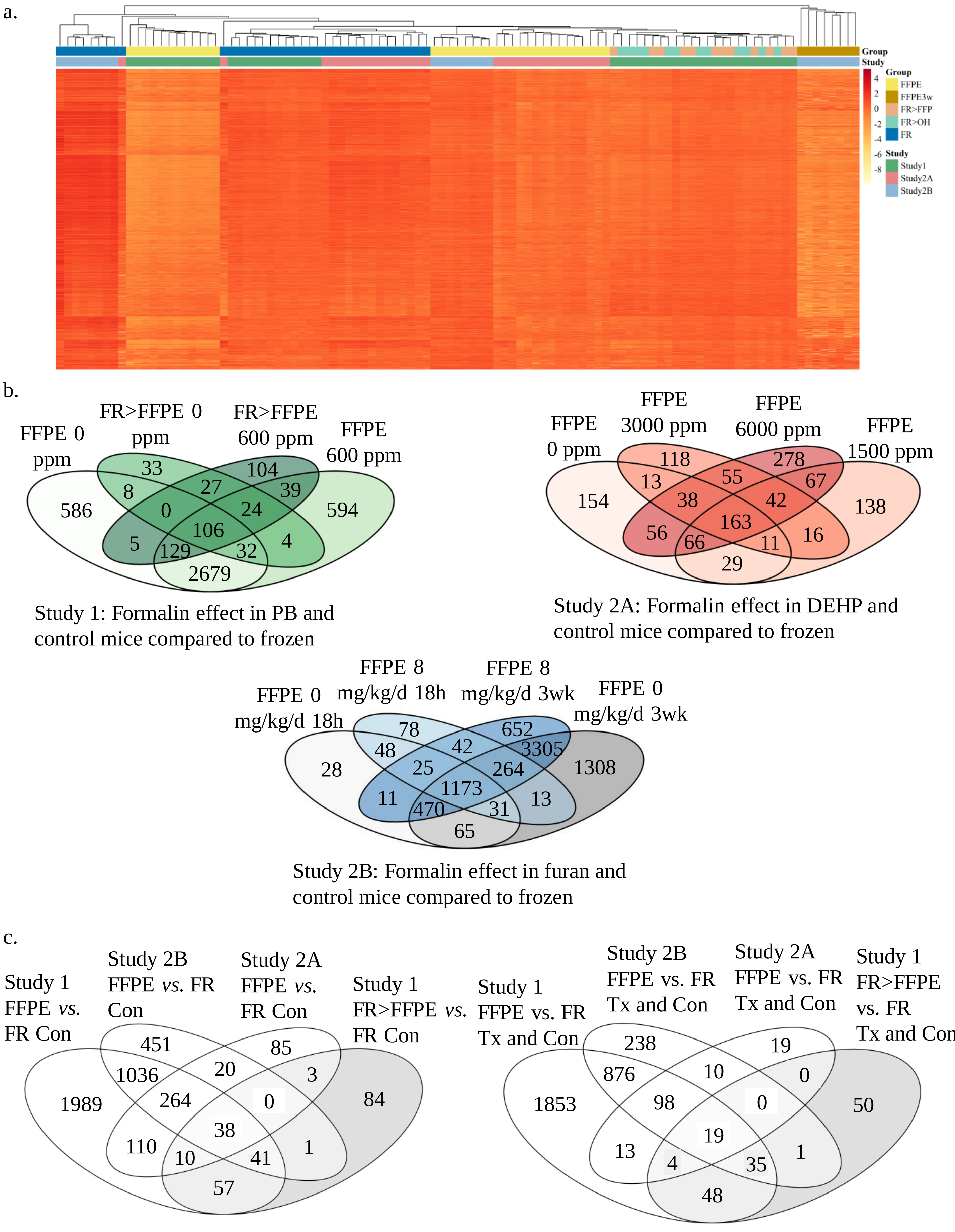

Supplementary Figure S6

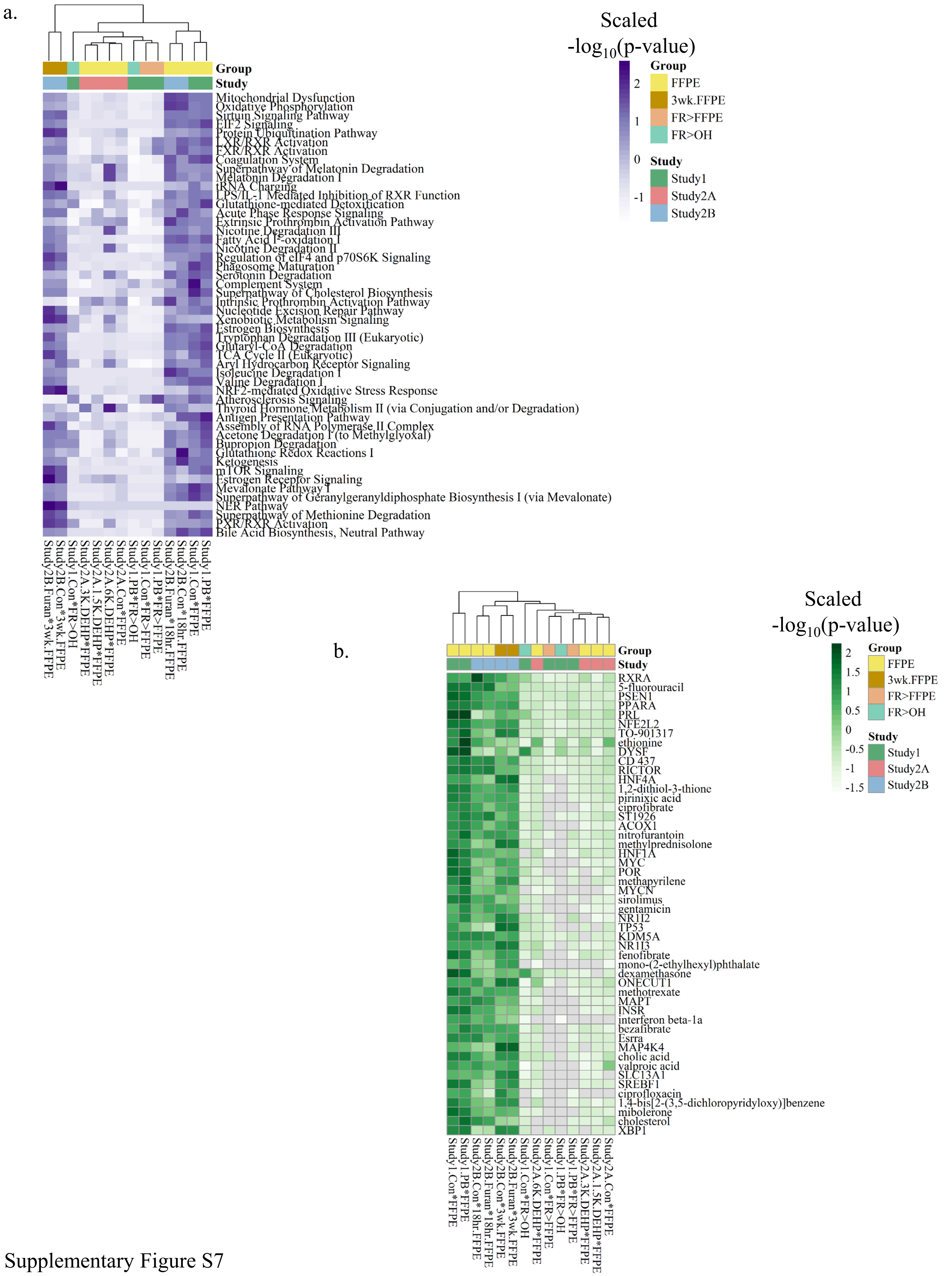

Supplementary Figure S7

a.

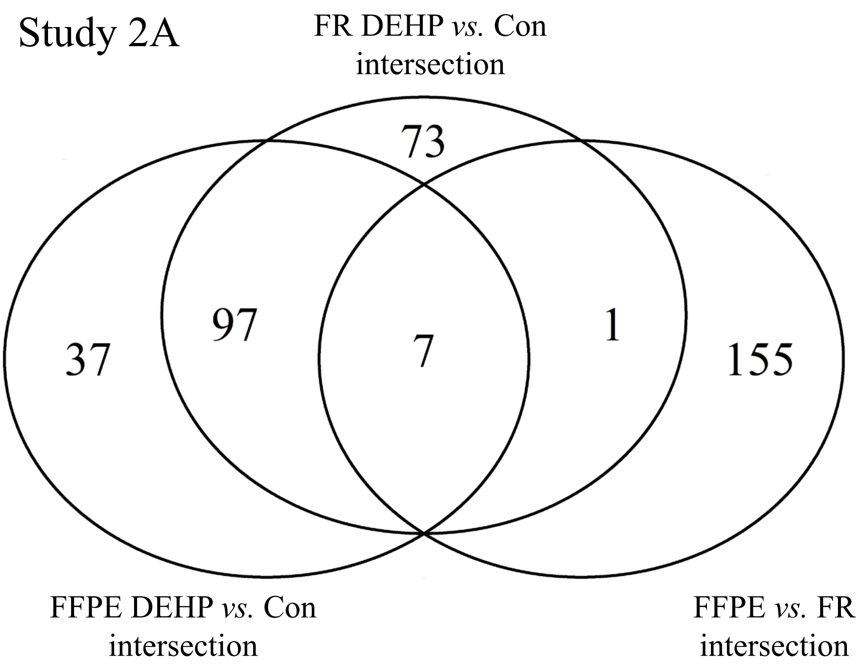

b.

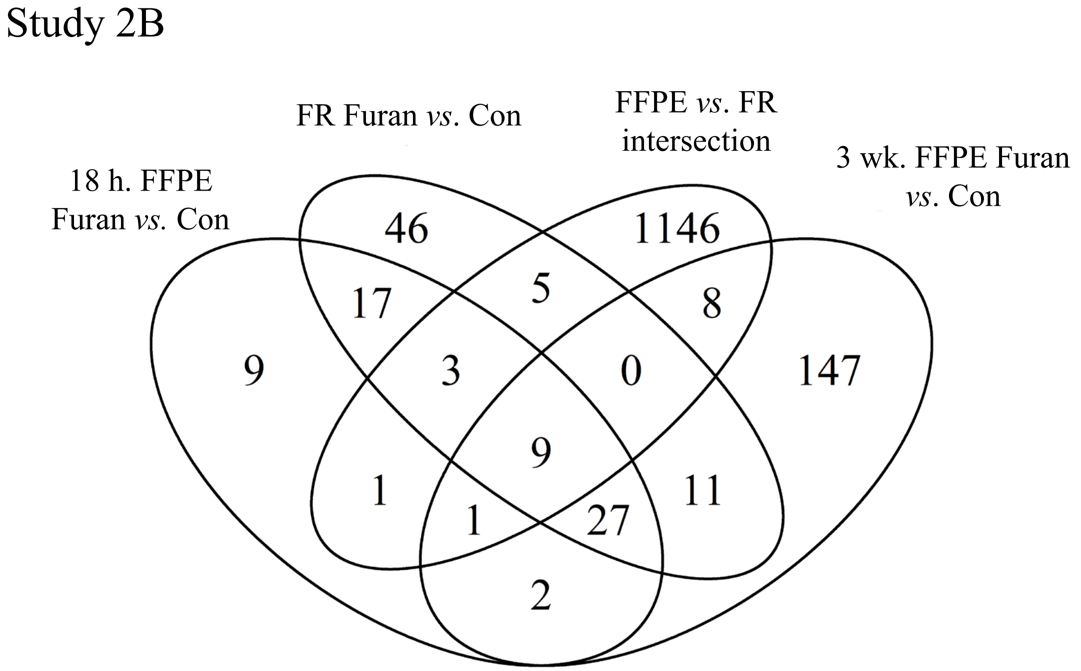

Supplementary Figure S8
