# Supplementary material for: Direct formalin fixation induces widespread transcriptomic effects in archival tissue samples
Source: Sci Rep. 2020 Sep 2;10:14497. doi: 10.1038/s41598-020-71521-w (PMC7468282; doi:10.1038/s41598-020-71521-w)
Supplement: Supplementary file 6 [file 41598_2020_71521_MOESM6_ESM.docx]

**SUPPLEMENTARY DATA**

Supplementary data located in five files. File 1 contains supplementary data tables referenced in the manuscript. Files 2-4 contain additional data tables including RNA-sequencing pre and post alignment quality metrics, raw and normalized gene counts, and differentially expressed gene tables. The supplementary figure file contains the supplementary figures referenced in the manuscript.

**SUPPLEMENTARY FIGURE LEGENDS**

**Supplementary Figure S1**. Study 1- Heatmap and hierarchical clustering of mean-centered and scaled count data from significant differentially expressed genes in any preservation group (FFPE, FR>FFPE, or FR>OH) *vs*. frozen with FDR-adjusted p-value <0.05 and absolute fold change ≥2 in Partek Flow GSA. Hierarchical clustering was based on average Euclidean distance. Abbreviations: FR, frozen; FR>OH, frozen first then fixed in 70% ethanol prior to paraffin-embedding; FR>FFPE, frozen first then fixed in 10% NBF for 18-24 hours prior to paraffin-embedding; FFPE, directly fixed in 10% NBF for 18-24 hours or 3 weeks prior to paraffin-embedding; PB, phenobarbital; Con, control.

**Supplementary Figure S2**. a) Study 1 - Heatmap of top 50 significant canonical pathways enriched by formalin-altered genes relative to frozen (FR) across preservation groups. b) Study 1 - Heatmap of top 50 upstream regulators predicted from formalin-altered genes relative to frozen (FR) across preservation groups. Significance determined by Fisher’s Exact Test with a p-value <0.05 in IPA.

**Supplementary Figure S3.** Study 1 - Overlap in formalin effect (including the preservation effect intersection) with phenobarbital (PB) response genes compared to vehicle control (Con).

**Supplementary Figure S4**. Study 1 - Heatmap and hierarchical clustering of mean-centered and scaled count data from top 50 significant phenobarbital (PB)-induced genes compared to vehicle control (Con) across preservation groups identified by FDR-adjusted p-value <0.05 and absolute fold-change ≥2 in Partek Flow GSA. Hierarchical clustering was based on average Euclidean distance. Darker red colors indicate higher scaled counts while darker gray colors indicate lower scaled counts. Abbreviations: FR, frozen; FR>OH, frozen first then fixed in 70% ethanol prior to paraffin-embedding; FR>FFPE, frozen first then fixed in 10% NBF for 18-24 hours prior to paraffin-embedding; FFPE, directly fixed in 10% NBF for 18 prior to paraffin-embedding; TX designates chemical treatment with PB, phenobarbital; Con, control.

**Supplementary Figure S5.** Study 1 - Expression pattern of top 10 phenobarbital (PB)-induced differentially expressed genes. Within preservation group comparisons of PB (▲) *vs*. vehicle control (Con, ●) resulted in significant differences in gene counts across most highly impacted genes (Wilcoxon rank sum test, p-value <0.01), as identified in transcriptome wide analyses. Across preservations within chemical treatment or vehicle control, ethanol (FR>OH), freezing first then formalin fixation (FR>FFPE), and direct formalin fixation (FFPE) followed by paraffin-embedding resulted in reductions of gene counts for all preservations but were not significant (Wilcoxon signed rank test, p-value <0.05). P-values were adjusted for multiple comparisons using Holm correction. The lower and upper hinges of the boxplot correspond to the first and third quartiles (the 25th and 75th percentiles). The upper whisker extends from the hinge to the largest value no further than 1.5 *(distance between the first and third quartiles or inter quartile range-IQR). The lower whisker extends from the hinge to the smallest value at most 1.5 * IQR of the hinge. Data beyond the end of the whiskers are individual outlying points.

**Supplementary Figure S6**. Study 1 and 2 - Formalin effects on differentially expressed genes (DEGs) following treatment with vehicle control or a reference chemical in mouse liver and the overlap of DEGs between dose groups within each study. a) Heatmap and hierarchical clustering of mean-centered and scaled log_2_ count data for significant differentially expressed genes present across all samples from Study 1, 2A, and 2B. Hierarchical clustering was based on average Euclidean distance. b) Study 1 involving mice exposed to PB or Con; Study 2A involving mice exposed to multiple concentrations of DEHP or vehicle control; and Study 2B involving mice exposed to furan or vehicle control. c) Venn diagram displaying overlap in significant 18 h. fixed formalin effected (FFPE *vs*. FR) genes for vehicle controls across Study 1, Study 2A and Study 2B with contrast to FR>FFPE *vs*. FR DEGs or for the intersection of vehicle controls and chemical treated samples. Significant genes identified by FDR-adjusted p-value <0.05 and absolute fold-change ≥2 in Partek Flow GSA. Abbreviations: FR, frozen; FR>OH, frozen first then fixed in 70% ethanol prior to paraffin-embedding; FR>FFPE, frozen first then fixed in 10% NBF for 18-24 hours prior to paraffin-embedding; FFPE, directly fixed in 10% NBF for 18-24 hours or 3 weeks prior to paraffin-embedding; Tx, chemical treatment (Study 1: PB, Study 2A: DEHP, and Study 2B: Furan); PB, phenobarbital; Con, vehicle control; DEHP, Di(2-ethylhexyl)phthalate.

**Supplementary Figure S7**. Study 1 and 2 - Retrospective analysis to confirm preservation-related effects of formalin fixation compared to fresh-frozen tissues, mapped to a) top 50 significant enriched canonical pathways and b) top 50 upstream regulators in common across present and two independent studies in mouse liver. Significance determined by Fisher’s Exact Test (p-value <0.05).

**Supplementary Figure S8.** Study 2 - Formalin effects across Studies 2A and 2B, showing minimal interference with detection of chemical treatment response. a) Study 2A overlap in formalin effect identified as the intersection of FFPE *vs*. FR across all Di(2-ethylhexyl)phthalate (DEHP) concentrations with the intersection across all doses of DEHP response genes compared to vehicle control (Con) in FFPE samples and FR samples. b) Study 2B overlap in formalin effect from 18 h. and 3 wk. FFPE *vs*. FR with the intersection across all preservations of furan response genes compared to vehicle control (Con).
